# What Is Going Through Your Mind? Thinking Aloud as a Method in Cross-Cultural Psychology

**DOI:** 10.3389/fpsyg.2018.01292

**Published:** 2018-08-13

**Authors:** C. Dominik Güss

**Affiliations:** Department of Psychology, University of North Florida, Jacksonville, FL, United States

**Keywords:** thinking aloud, verbal protocols, transitional probability, cross-cultural, culture, complex problem solving

## Abstract

Thinking aloud is the concurrent verbalization of thoughts while performing a task. The study of thinking-aloud protocols has a long tradition in cognitive psychology, the field of education, and the industrial-organizational context. It has been used rarely in cultural and cross-cultural psychology. This paper will describe thinking aloud as a useful method in cultural and cross-cultural psychology referring to a few studies in general and one study in particular to show the wide applications of this method. Thinking-aloud protocols can be applied for (a) improving the validity of cross-cultural surveys, (b) process analysis of thoughts and the analysis of changes over time, (c) theory development across cultures, (d) the study of cultural meaning systems, and (e) individual as well as group level analyses allowing hypothesis testing cross-culturally. Limitations of the thinking-aloud method are also discussed.

## Introduction

Thinking aloud is the concurrent verbalization of thoughts while performing a task (Ericsson and Simon, 1993). When this method is applied, participants are asked to spontaneously report everything that goes through their minds while doing a task, and they are instructed not to interpret or analyze their thinking. *Verbal protocol* is another term often used as a synonym for thinking aloud. Verbal protocols can be concurrent (thinking aloud) or retrospective, referring to short reports after the completion of a task.

The study of thinking aloud and of verbal protocols has a long tradition in psychology. It can be traced back to Wilhelm Wundt’s technique “Selbstbeobachtung” (self-observation, also often called introspection; Wundt, 1888). Wundt asked participants in his experiments to look inward, pay attention to their inner thought processes, and describe them in detail. Wundt perceived the inner experience, the flow of consciousness, as the core topic of psychology. He saw self-observation as an appropriate method for studying this flow of consciousness when it occurred under controlled conditions in the laboratory. Some researchers criticized the method, believing that self-observation would interfere with the thought process and, thus, would not show the real thought process itself, but rather an interpretation of the thought process (Ericsson and Crutcher, 1991).

The thinking-aloud method was heavily criticized by behaviorists, as they assumed cognitive processes, such as memory, could not be studied scientifically. As Watson (1925) expressed, “The behaviorist never uses the term *memory*. He believes that it has no place in an objective psychology” (p. 177). The thinking-aloud method became popular again after the influence of behaviorism diminished in mainstream psychology and cognitive psychology became the dominant paradigm. Newell and Simon (1972), for example, asked participants to think aloud while solving particular problems. Rather than investigating whether a person solved a problem or not, their focus was on the process of human reasoning while solving problems. From these thinking-aloud protocols, they derived the computer-simulated model “General Problem Solver.”

A study conducted by Ericsson and Chase (1982) on exceptional memory showed that a student could increase his digit span from 7 (e.g., 3-5-1-3-7-8-2), the average number of digits a person is able to remember, to 80 digits by training 1 h per day, three to five times a week for 20 months. Retrospective verbal protocols showed that the participant used specific mnemonics to help him remember. One mnemonic was to group the digits together in meaningful units, which is called chunking. For example, the three digits 3 5 1 could be grouped together as one chunk of “3 min 51 s – close to world record mile time,” which, if the participant was a long-distance runner, as this participant was, would make sense and, thus, would be easier to remember. Since the publication of Ericsson and Chase’s work, thinking aloud has been recognized as an acceptable and even essential method in the study of human cognition.

## The Wide Application of the Thinking-Aloud Method

Thinking aloud as a scientific method has been used in many other disciplines, showing the relevance and applicability of this method. Not only researchers studying cognition (e.g., Fleck and Weisberg, 2004; Hölscher et al., 2006; Malek et al., 2017), but also researchers studying education (e.g., Cummings et al., 1989; van den Bergh and Rijlaarsdam, 2001; Bannert, 2003; Kesler et al., 2016), text comprehension using computer based tools (Muñoz et al., 2006; Van Hooijdonk et al., 2006; Wang, 2016), discourse processing (Long and Bourg, 1996), software engineering (Hughes and Parkes, 2003), psychology and law (Santtila et al., 2004), sport psychology (e.g., Samson et al., 2017), and business management (Isenberg, 1986; Premkumar, 1989; Hoc, 1991) have applied the thinking-aloud method. In a similar form, thinking aloud has also influenced the fields of counseling and clinical psychology, for example, in the assessment of automatic thoughts as part of cognitive therapy in depression (e.g., Meichenbaum, 1980; DeRubeis et al., 1990).

## Reliability and Validity of Thinking-Aloud Protocols

Following positivism, reliability and validity are central to research. Reliability of thinking-aloud data refers to consistency, the ability to collect the same data at a different time. In order to get reliable data, a clearly understandable, tape or digitally recorded thinking-aloud protocol is necessary, which requires control of the experimental situation. Problems related to transcribing and especially to coding have to be minimized, and, ideally, transcription should be done by native speakers of the participants’ language. The first step of coding is the segmentation of the whole protocol, i.e., the creation of separate meaningful units, depending on the research questions of interest. Usually those statements are in the form of clauses or sentences; these sentences do not need to be complete, necessarily, as participants use colloquial language. The second step refers to the coding of the segments. A detailed coding system and thorough training of coders can increase reliability, resulting in higher inter-coder reliability. This reliability is sometimes described in percent of agreement, but preferably should be described in Cohen’s Kappa or intraclass correlation coefficients. According to Fleiss (1981), a Kappa value over 0.75 is excellent, between 0.60 and 0.75 is good, and a Kappa between 0.40 and 0.60 is fair. One problem that may be encountered during coding is coder biases or expectations, as can occur when the coders are aware of the hypotheses to be tested, for example. Ideally, then, the coders should not know about the research hypotheses. Also, probable biases or expectations can be acknowledged early to increase trustworthiness of the coding process and, consequently, of the data.

Also at issue is the internal validity of a study. In the context of thinking aloud, the validity question is often framed as the reactivity question. Does the act of thinking aloud interfere with and change a person’ cognitive processes while performing the task? Ericsson and Simon (1993) argued that it did not, citing many studies and stating that as long as the instruction was clear, i.e., that participants should say out loud everything that went through their minds, thinking aloud did not alter the sequence of thoughts. However, prior consideration should be given to the way instructions are to be conveyed to participants. An instruction from a facilitator to “keep talking” while the participant performed a task probably would not disrupt the thought process, though an instruction requiring explanation from the participant, like “Tell me why you did this,” would intervene in the cognitive process by triggering a specific answer to explain an action. If verbal protocols are asked from participants after completion of tasks, it is preferable if verbalization almost immediately follows the task. Generally, a concurrent thinking-aloud protocol has higher validity than a retrospective report, particularly when the task takes a long time to complete.

One way to ensure reliability and validity and to determine whether thinking aloud influences a cognitive process is to create two groups: an experimental group that receives instruction to think aloud, and a control group that does not receive such instruction.

## Qualitative Evaluation Criteria: Trustworthiness of Thinking-Aloud Protocols

Researchers conducting qualitative studies use different criteria to evaluate the quality of their research. Whereas quantitative psychologists try to discover general universal laws, qualitative researchers try to understand participants’ “lived experience” (Hesse-Biber and Leavy, 2006, p. 62), assuming a socially constructed reality. Lincoln and Guba (1999) described four criteria guaranteeing the trustworthiness of the research: credibility, transferability, dependability, and confirmability. Credibility refers to how confident one can be regarding the truth of a study’s findings. One way to support credibility is to be open to the possibility of falsification and to conduct a “negative case analysis” (Hesse-Biber and Leavy, 2006, p. 63), i.e., to include cases that contradict or are not in line with the conclusions drawn so far. It speaks for the researcher if he or she is willing to include those cases in the analysis and, as a result, is able to revise the previously drawn conclusions. A second method to support credibility is triangulation. Triangulation can refer to the use of several methods or several sources of information to investigate the same research question. Thinking aloud, for example, could be combined with post-experiment interview or survey data. Triangulation can also refer to different investigators working on the same data set. This is especially relevant for cross-cultural studies and analyses of thinking-aloud protocols. The underlying assumption of triangulation is that it provides a fuller and more credible picture of the phenomenon. Extended experience in the environment can also increase credibility, and it is especially important for cross-cultural psychologists to learn about the other culture and learn the language in order to get a deeper understanding of the utterances made by the participants in the thinking-aloud protocols.

Transferability refers to the application of the findings to other contexts or other people. Quantitative researchers pursue random sampling. Qualitative studies often include small sample sizes and pursue purposive sampling with the goal of getting a wide variety and range of information that can increase the transferability.

Dependability is the third criterion and refers to a study’s reliability. Confirmability, the fourth criterion, refers to the accuracy of findings, and to what extent they were influenced by the researcher’s biases. Researchers can increase both dependability and confirmability by journaling their experiences and biases and by engaging in dialog with other researchers early on in the research process. Participatory research and peer review (Willis, 2007) can also increase dependability and confirmability. In participatory research, the researcher presents initial conclusions of the study to the participants and actively involves them in the research process. For example, thinking-aloud protocols could be shown to and discussed with the participants and ambiguities in the protocol could be clarified. Peer review is similar to triangulation involving other researchers. In cross-cultural research, the ideal, as mentioned before, would be collaboration with a researcher from the target culture. It is recommended to involve other researchers early in the research process and to stay in continuous dialog with them about the research progress.

## Cross-Cultural Psychological Research Using Thinking-Aloud and Verbal Protocols

One goal of cross-cultural psychology is “the study of similarities and differences in individual psychological functioning in various cultural and ethnocultural groups” (Berry et al., 2002, p. 3). The thinking-aloud method, however, is rarely used in cross-cultural research. A search in PsychInfo, January 2018, with no time limitation showed 503 hits for the term “thinking aloud” used anywhere and 464 hits for the term “verbal protocols” used anywhere. Only six peer-reviewed journal articles were found for the combination of the word “thinking aloud” or “verbal protocol” anywhere with either one of the two keywords “culture” or “cross-cultural.”

Luria (1976) studied reasoning, among other cognitive processes, in Central Asia, comparing illiterate peasants (the term used by Luria), barely literate kolkhoz farm workers, and young people with a few years of schooling. He used an interview technique to investigate the thought processes of the participants. He presented participants with syllogisms such as the following: “Cotton grows well where it is hot and dry. England is cold and damp. Can cotton grow there or not?” (p. 107).

Results showed that illiterate participants who had no formal education had difficulties solving the syllogisms. Luria was not only interested in the outcome, how many of the participants of each group could solve the syllogism correctly, but even more so in their reasoning, how they interpreted the syllogism. The illiterate participants interpreted the syllogisms on the basis of their experiences in a concrete way and did not show abstract thinking. Only the analysis of participants’ thought processes allowed Luria to answer the question of *why* illiterate participants had difficulties interpreting the syllogisms.

The following is part of a short conversation the interviewer had with a 37-year-old illiterate villager who was presented with the cotton syllogism. It is, however, more an interview than a mere thinking-aloud protocol.

Interviewer: “Cotton can grow only where it is hot and dry. In England, it is cold and damp. Can cotton grow there?”
Participant: “I don’t know.”
Interviewer: “Think about it.”
Participant: “I’ve only been in the Kashgar country. I don’t know beyond that.”
Interviewer: “But on the basis of what I said to you, can cotton grow there?”
Participant: “If the land is good, cotton will grow there, but if it is damp and poor, it won’t grow. If it’s like the Kashgar country, it will grow there too. If the soil is loose, it can grow there too, of course” (Luria, 1976, p. 108).

Luria also used grouping tasks where participants were presented with several objects and had to find, which ones belong together and which ones did not. This task assesses categorical classification. The following is the response of a 60-year-old illiterate peasant who was shown pictures of a hammer, a saw, a log, and a hatchet.

They all fit together! The saw has to saw the log, the hammer has to hammer it, and the hatchet has to chop it. And if you want to chop the log up really good, you need the hammer. You can’t take any of these things away. There isn’t any you don’t need (Luria, 1976, p. 58).

This thinking-aloud statement related to the classification task shows the participant’s situational thinking. The participant does not classify the objects into a more abstract category, but refers to their “practical utility” (p. 59). Similar studies on formal and informal education and its influence on problem solving, reasoning, or intelligence were reported by Scribner (1979) and Scribner and Cole (1981), who also instructed participants to verbalize their thoughts when solving certain cognitive tasks. These studies show that thinking aloud can tap into information that cannot be analyzed by other methods alone, explaining the differences or accessing the nuances usually not revealed through other forms of data gathering.

### Cultural Meanings 1: Improving the Validity of Cross-Cultural Surveys Using Thinking Aloud

Raitasalo et al. (2005) used the thinking-aloud method in a cross-cultural study in Finland, Germany, Italy, and the Netherlands to investigate cultural differences in answering survey items. The survey focused on alcohol use: frequency of drinking, quantity of drinking, frequency of drunkenness, and the context of drinking in the last 12 months. For our purposes, the major finding of interest is cross-cultural differences related to the understanding of the survey questions. We can conclude from this study that allowing participants in cross-cultural studies to verbalize or write down their thoughts when answering Likert-scale survey questions could show the researcher(s) how the participants understand the questions and which cultural meanings participants attribute to these questions. Thinking aloud can also point out the interpretations participants give to the survey questions.

To illustrate this point, I would like to quote two survey questions used in studies published in the *Journal of Cross-Cultural Psychology*. The first one refers to Keller et al. (2006), who used the Family Allocentrism Scale (Lay et al., 1998) as one of their measurements. Lay et al. tested for response bias and conducted item analyses with western and eastern samples when they developed their survey. One item of this scale is “My family’s opinion is important to me.” Thinking aloud of participants from different cultural groups regarding this question could be especially beneficial in the first stages of scale development and could reveal (a) if participants think of specific family opinions, (b) if so, which ones they are referring to when answering this question, and (c) who and what defines family: a nuclear family; an extended family with grandparents, uncles, aunts; only one caregiver; or if family is interpreted as only the participant, the individual. One western participant could express, for example: “No, their opinion is not important to me when they want to tell me which clothes I should wear.” Another participant could say, “Sure, I listen to their advice regarding my future major at the university. After all, they will support me.” Another participant might say, “I listen to my mom, because she understands me, but not to my dad, and certainly not to my brothers.” These different answers show that participants understand the question in different ways and participants’ answer choices depend on what they are thinking of at the time. One could specify the question, for example: “My mother’s opinion regarding my professional future is important to me.”

A second example is an item Hershey et al. (2007) used studying retirement planning in the Netherlands and in the United States, a single-item indicator for perceived savings adequacy: “I am saving enough for retiring comfortably.” A participant in Germany might choose, “1-strongly disagree,” thinking aloud, “I do not need to save and I did not save, because I always paid into the social retirement system and I am guaranteed a retirement from the government.” An Indian participant might also choose, “1-strongly disagree,” thinking aloud, “I do not need to save, because I have four children and they will take care of me; one of them is even a computer programmer in Hyderabad.” Additionally, a Filipino might also choose, “1-strongly disagree,” but say, “No matter how hard I try, I will never be saving enough for retirement, there is no well-functioning system of retirement here. We grow old, we stay with our family, we are loved.” Even if the German, Indian, and Filipino have the same survey answer, all indicating that they are not saving for retirement, their thinking-aloud statements show that the underlying reasons for their responses are quite different. The researcher could use those thinking-aloud data to specify the question and perhaps to develop further questions to lessen misinterpretation, garner more accurate responses, or even to be more sensitive to participants’ culture. Possible modified items could be “The government is supporting retired people adequately.” And “I can rely on my family to support me financially when I retire.”

The use of thinking aloud and verbal protocols can be especially helpful when surveys try to assess sensitive topics, meaning topics subject to bias and social desirability, and to those that attempt to be respectful to the context and the larger dimensions of the culture. Edwards et al. (2005), for example, conducted a study on condom use as a preventive measure for HIV/AIDS. They collected thinking-aloud protocols of sex workers problem solving a simulated task. The goal was to improve a sexual behavior survey instrument. The thinking-aloud data helped the authors to improve comprehension of the instrument and to reduce social desirability, providing appropriate terms and cues for aiding recall, improving the establishment of trust with participants, and creating a sense of cultural competence and credibility from the researchers. Vreeman et al. (2014) transcribed and coded cognitive interviews regarding HIV with pediatric caregivers in Kenya in order to further develop and adapt survey items to this cultural context.

Cross-cultural psychologists have a multitude of quantitative methods to increase reliability and validity of survey instruments used in cross-cultural research (for an overview, see van de Vijver and Leung, 1997; van de Vijver and He, 2017). The thinking-aloud method is an additional method that can be used to improve the reliability and validity of self-report instruments (Sudman et al., 1996).

### Cultural Meanings 2: Thinking Aloud Allows for the Study of Cultural Meaning Systems Beyond the Sentence Level

The previous paragraphs referred to cultural meanings attributed to specific survey items on the sentence and phrase level. Thinking-aloud data can be also analyzed more broadly regarding the meanings expressed by participants going beyond the sentence level. A multitude of other qualitative methods, such as consensual qualitative research methodology (Hill et al., 1997; Güss et al., 2018) or grounded theory (Glaser and Strauss, 1980) can be applied to analyze thinking-aloud protocols for meanings expressed by participants of various cultural and ethnic groups. As Smagorinsky (2001) pointed out, “from a cultural perspective a verbal protocol represents the speaker’s cultural conception of the word” (p. 235) and gives insight into his or her cultural world. Needless to say, analysis of such protocols necessarily requires coders from the participants’ respective cultures or coders who are multiculturally competent – not only knowledgeable about other cultures, but deeply aware of their own biases and prejudices.

### Concrete Examples of Thinking-Aloud Data Analysis From One Cross-Cultural Study

A study by Güss et al. (2010) illustrates the different options for data analysis using thinking-aloud protocols. The study was conducted in Brazil, Germany, India, Philippines, and the United States with over 500 participants. They were instructed to think aloud while working on two computer-simulated problem -solving tasks. One of the tasks was a computer simulation in which participants took the role of a fire-fighting commander who had to protect three cities and forest from approaching fires. Participants always spoke in their native languages when they thought aloud. However, Indian and Filipino participants often spoke in English. We encouraged participants to use the language they were most comfortable using to minimize potential influences of thinking aloud on the problem-solving process. All the thinking-aloud protocols were tape-recorded, transcribed, and coded. Student volunteers in every country were trained how to transcribe and code the protocols. During the training, the coding system was explained and defined, examples were given, coding was practiced, and the differences between the subcategories were discussed.

Each thinking-aloud protocol was transcribed into Microsoft Excel, so that every statement expressing an idea unit filled one cell. The following example has two different idea units and was therefore transcribed into two cells: “I send truck 5 to city 1 // and then I will clear the forest.” Statements were then coded according to the problem-solving stages. The coding system was initially created following the western stage model of problem solving: problem identification, goal definition, information gathering, mental model building, planning of solutions, prediction of further developments, decision-making, action, evaluation of outcome, and modification of strategic approach (e.g., Bransford and Stein, 1993; Dörner, 1996). The system was then modified to account for other statements made by participants. These statements referred to emotions and self-descriptions. The final coding system consisted of 21 categories that were summarized in 8 main categories (Güss et al., 2010).

### Testing Theories Across Cultures Using Thinking Aloud

**FIGURE 1:**
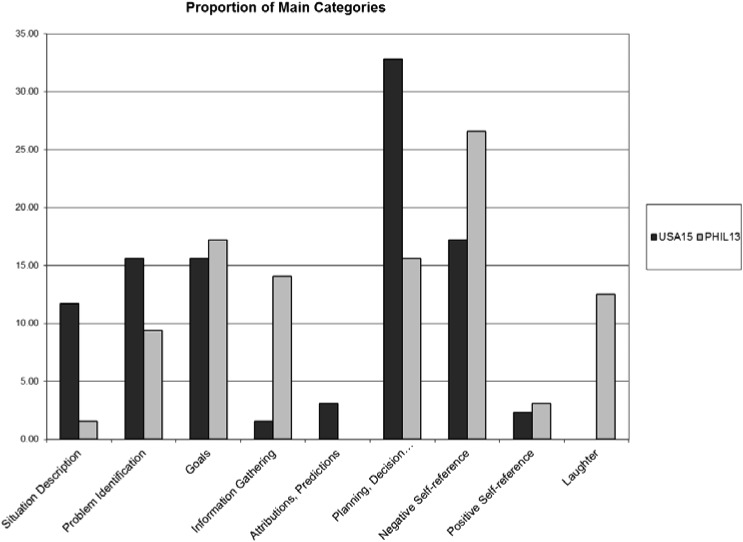
Proportions of categories expressed in thinking aloud for U.S. and Filipino participant (excluding other statements).

**Table 1** contains verbatim parts of participants’ thinking-aloud protocols and includes statements from one U.S. participant (USA15) and one Filipino participant (Phil13). The coding is also indicated (the full coding system is available upon request). These data can be used to test specific hypotheses. Based on a literature review (e.g., Nisbett, 2003), one hypothesis could refer to a more problem-centered and solution/action-oriented focus on problem solving for U.S. participants and a more context-centered focus for Filipino participants. The frequency of categories can be counted and either absolute or relative frequencies can be shown. **Figure 1** shows relative frequencies as the time required to complete the thinking-aloud protocols and the number of statements for the U.S. and Philippine participants differed.

**Table 1:** Coding and idea unit examples transcribed from a study using the thinking-aloud method.

**USA15**		
Send these guys walking around the houses to put some fires out around there	PlanDM	Planning, decision making, and action
Huh?	Info	Gathering of information
Send the helicopter over to the right	PlanDM	Planning, decision making, and action
Because its getting pretty close to the rich people	PlanDM	Planning, decision making, and action
Oaauhuh	SR-	Negative self-reference
Guess Johnny put out some fires still	AT_Pred	Attributions and predictions
Up at the top left or middle left	SD	Situation description
Don’t want to move that guy because the fire pops up in the middle then	PlanDM	Planning, decision making, and action
So still going there	PlanDM	Planning, decision making, and action
I need a helicopter and stuff extinguish the fire down there	GO	Formulation of goal
Okay, one is moving that way	SD	Situation description
So it’s probably not going to hit him	AT_Pred	Attributions and predictions
I mean, look at it when its moving	O	Other
Go southeast to the…	PlanDM	Planning, decision making, and action
Still trying to put out the fire	GO	Formulation of goal
It’s getting closer to the city	PI	Problem identification
**Phil13**		
I forgot already the commands.	SR-	Negative self-reference
Click this	GO	Formulation of goal
Go to the units	GO	Formulation of goal
Click again	GO	Formulation of goal
And that’s it!	SD	Situation description
Oh no, there again	SR-	Negative self-reference
Goal	PlanDM	Planning, decision making, and action
Extinguish	PlanDM	Planning, decision making, and action
Click again and then extinguish	GO	Formulation of goal
I am good.	SR+	Positive self-reference
(Laughs)	L	Laughter
Click again	GO	Formulation of goal


The distribution of the problem-solving categories of the complete thinking-aloud protocols shows which categories were used frequently and which ones were not. The most frequent category expressed by the U.S. participant (USA15) was planning, decision-making, and action – roughly one-third of all statements. For the Filipino participant (Phil13), the most frequent category, expressed in more than a quarter of all statements, was negative self-reference (SI). The distribution of the categories differed significantly between the U.S. and Filipino participant, χ^2^(169) = 200.27, *p* = 0.05. The U.S. participant showed relatively more situation description, PI, attributions and predictions, and planning, decision-making, and action. The Filipino participant expressed relatively more information gathering, negative SIs, and laughter. The two participants’ data support the hypotheses. Indeed, results indicated a dominance of categories related to problem identification and problem solution for the U.S. participant and a dominance of information gathering to understand the problem context and less solution-focus for the Filipino participant.

The analysis here refers only to thinking-aloud protocols of two individuals. The same analysis could be conducted for averages of thinking-aloud protocol categories among different cultural groups. Especially when referring to cross-cultural differences and when claiming reliable cross-cultural differences, then the data should be compared at the group level rather than individual level. In fact, a comparison of over 400 Brazilian, German, Filipino, Indian, and U.S. participants’ thinking-aloud protocols shows significant cross-cultural differences among exactly these problem-solving categories with medium to large effect sizes (Güss et al., 2010).

### Testing Cross-Cultural Generalizability of Psychological Theories Developed in Western Societies Using Thinking Aloud

The analysis of the thinking-aloud protocols can also be used to test theories that were developed in western industrialized countries for their cross-cultural validity. The dominant theory on problem solving developed in the United States (e.g., Bransford and Stein, 1993) and Europe (e.g., Dörner, 1996) suggests that problem solvers go through certain stages while solving problems. These stages are clarification of goals, gathering information, prediction of further developments, planning, decision-making, action, and evaluation of effects. In the Güss et al.’s (2010) study, many of these stages were indeed found in the thinking-aloud protocols of participants in all five countries. What the western stage model did not consider, however, were statements referring to negative and positive emotions and statements referring to negative and positive self-evaluations (e.g., “I will never be a good fire fighter”). Our data from the five countries showed that problem solving is not solely a cognitive process, but interacts with emotional and self-evaluative processes. The thinking-aloud data from the five countries support the existing stage model. On the other hand, they provide the basis to further develop the model and include emotional and self-evaluative processes.

### Testing Predictions and Differences in Performance Using Thinking Aloud

The thinking-aloud data can also be used as independent variables to test the influence on a dependent variable. One question relevant to the data of the U.S. and Filipino participant refers to which stages can predict performance in the fire simulation. Is it always the same stage or do these stages vary cross-culturally? Analyzing the demands of the simulation, i.e., the development of many fires and the requirement to extinguish them fast to avoid their spreading, indicates that the most crucial of the stages is planning, decision-making, and action. Although cross-cultural differences are expected in the frequency of the categories, it is likely that across cultures the same stages predict performance due to the specific task demands. USA15 protected 68.1% of the forest at the end of the simulations, Phil13 protected 53.1%. Correlations and regression analyses would allow testing those predictions referring to groups of participants. The correlation of performance in the simulation with the frequency of planning, decision-making, and action controlling for the overall number of statements made was *r* = 0.11, *p* = 0.04 (*N* = 349). This relationship, however, was not significant for the U.S. sample, *r* = 0.13, *ns* (*n* = 64), and only marginally significant for the Filipino sample, *r* = 0.22, *p* = 0.08 (*n* = 62).

The effect size (i.e., *r*) is smaller in the overall analysis across the United States and Filipino cultures than those within individual cultural samples. However, because of the difference in sample size, the correlation was only significant for the overall analysis. Thus, in this specific case, the result does neither support cultural universality nor cultural differences.

### Analysis of Transitions in the Process Using Thinking Aloud

The thinking-aloud data can be analyzed in more detail. One might ask, for example, if the Filipino participant’s laughter is a positive expression related to happiness and other positive emotions or if it is nervous laughter, a coping mechanism relieving negative emotions and tensions. Another question of interest could be related to cultural strategies in problem solving. What do participants do when they identify a problem – for example, a new fire spreading close to one of the cities?

These questions can be answered analyzing the transition probabilities between the stages, also called lag analysis (Bakeman and Gottman, 1986) or latent transition analysis (Lanza et al., 2005). The transitional probability (TP) from any category *x* to another category *y* is given by TP (*x* → y) = frequency (*xy*)/frequency (*x*).

**FIGURE 2:**
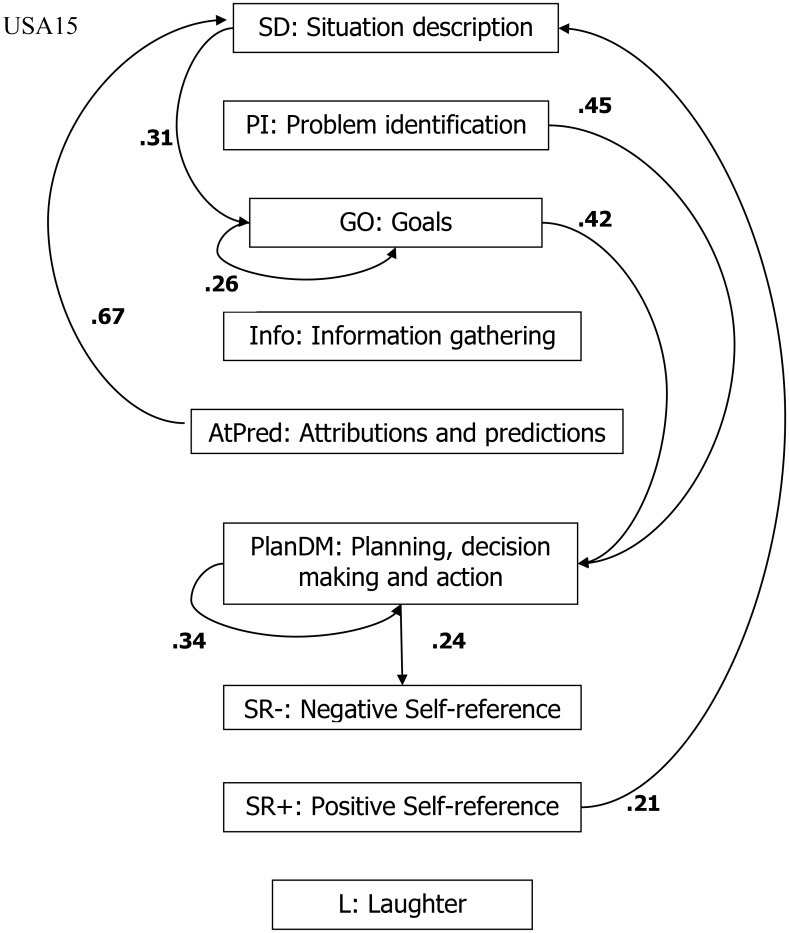
Lag analysis: most frequent transitions (excluding other statements) for USA15.

**FIGURE 3:**
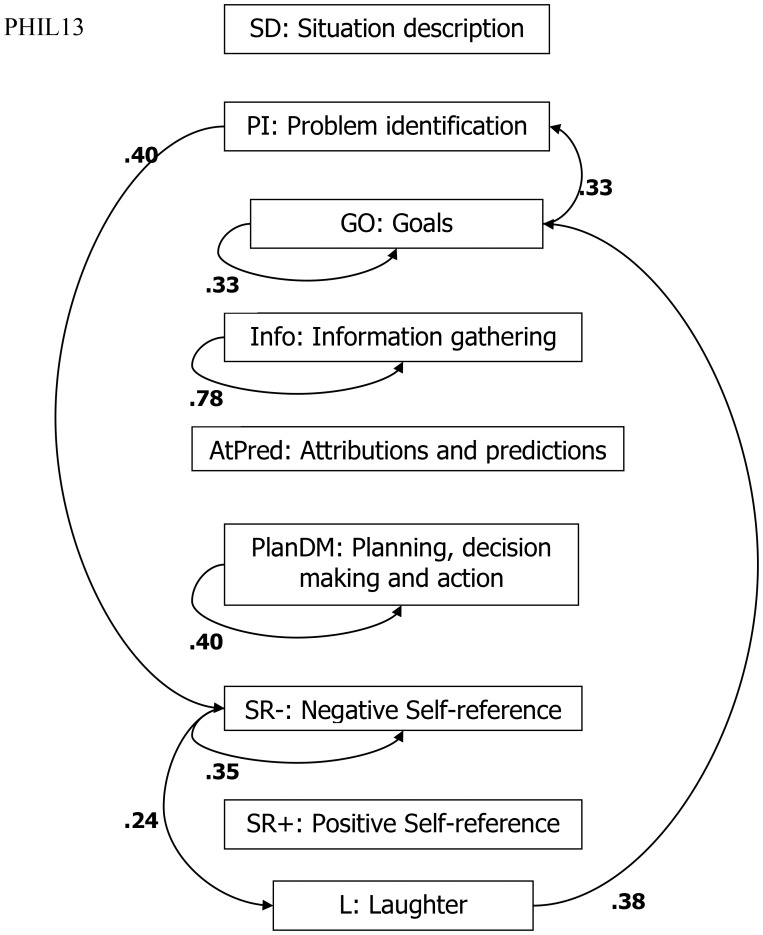
Lag analysis: most frequent transitions (excluding other statements) for Phil13.

We could examine the thinking-aloud protocols to discover what statements the Filipino participant made before laughing (L). What is the probability that laughing (*y*) follows negative self-reference statements (*x*)? Or, what statements were made by both participants after they identified a problem (PI)? This analysis can be quite tedious when done manually in long or multiple protocols, so we developed a computer program (Parise and Güss, 2006) that can read the coded files and give an output file with all the possible transitions. The most frequent transitions in the thinking-aloud protocols of USA15 and Phil13 are shown in **Figures 2, 3**. The figure for PHIL13 shows that laughter was preceded in 24% of all transitions by negative self-reference statements. This might indicate that laughter was used to cope with negative emotions and negative self-evaluations.

The question above about statements made after PI had to do with culture-specific ways of dealing with problems. The Filipino transitions showed that after PI, the most frequent reaction was a negative self-reference statement (40%). The U.S. participant reacted differently. In the figure showing the U.S. participant’s transitions, the most frequent transition from PI was to planning, decision-making, and action (45%). Whereas the Filipino reacted to PIs with negative emotions and self-evaluations, the U.S. participant proceeded right to the solution process.

The previous analyses referred to one Filipino and one U.S. participant. We also created a program (Edwards, 2018) that compiles all the transition frequencies of the 74 Filipino and the 67 U.S. participants. Although laughter happened almost six times more often in the Filipino sample, it was preceded by negative self-references in 24.6% in the U.S. sample and in 23.2% in the Filipino sample. The differences we discussed before regarding laughter were not found in the two overall cultural samples.

We also analyzed the thinking-aloud protocol transitions for all Filipino and all U.S. participants regarding the stages following PI. Overall, U.S. participants mentioned problem statements twice as often as Filipino participants. Whereas negative self-references followed PI in 14.3% in the U.S. sample, planning, decision-making, and action followed in 53.4% of all transitions. In the Filipino sample, negative self-references followed slightly more often, namely in 16.1% and planning, decision-making, and actions only in 14.4% of all transitions after PI. The tendency we discussed before to proceed with planning and decision-making after a problem is identified was also found in the U.S. sample overall.

The statistical significance of transition probabilities can be tested using chi-square tests and comparing the probabilities with the probabilities expected by chance. If several chi-square tests are run, alpha levels can be adjusted using Bonferroni to reduce Type I error. The example given refers to two-way timetables, i.e., only one stage following another stage was analyzed. The sequences are also called a Markov chain. Depending on the theoretical question, it is also possible to investigate patterns larger than a combination of two, for example, a sequence of PI – negative SR- – planning, decision-making, and action (PlanDM). Statistical methods can help to determine the order of the Markov chain and to test the homogeneity of the transition frequencies (Gottman and Roy, 1990; Bakeman and Quera, 1995; Muthén and Muthén, 2017).

### Studying Changes Over Time Using Thinking Aloud

Thinking-aloud data allow another analysis of the process as well. A researcher might be especially interested in changes that happen over time. Special hypotheses regarding changes in the problem-solving process can be formulated. During the 12 min of the fire simulations, participants might adapt to specific demands of the simulation. Initially, for example, there are no fires, and the participant has time to get familiar with the situation. During that stage, definition of goals might be important: “What do I want to do and achieve?” Toward the middle of the simulation, when several fires are burning, decision-making, and action might be the necessary and dominant stage. Toward the end, a participant might reflect on what he or she has accomplished.

**FIGURE 4:**
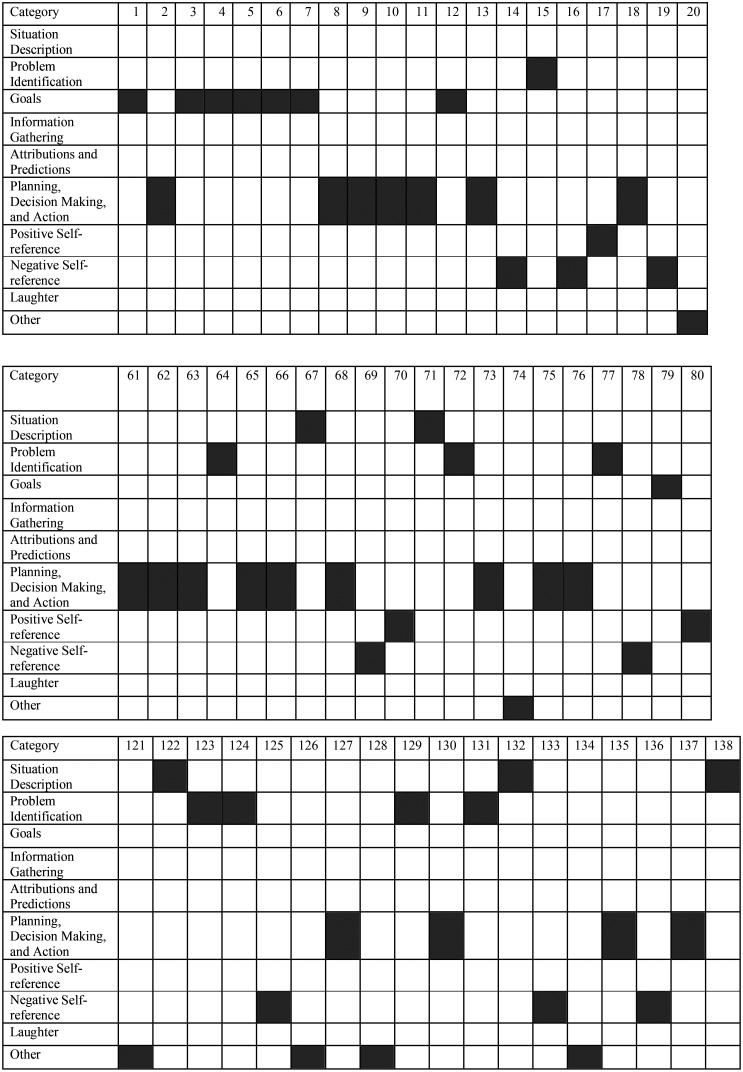
Coded thinking-aloud statements from the beginning, middle, and end of the simulation.

**Figure 4** shows the first 20 coded statements at the beginning of the fire simulation, 20 coded statements made in the middle of the simulation, and the last 20 coded statements made at the end of the simulation. Due to space limitations, only the process of the U.S. participant (USA15) is shown. As expected in the hypotheses, initially (codes 1–20) the participant verbalized many goal statements and then moved into planning, decision-making, and action followed by some self-reference statements. In the middle of the simulation (codes 61–80), planning, decision-making, and action was the dominant stage. Some statements referred to problem identification, situation description, and self-references. Toward the end (codes 121–138), however, there is no dominant stage. Every category expressed had a frequency of three or four.

Cross-cultural comparisons could reveal that participants from other cultures follow a different approach. They might, for example, not plan ahead and start with goals, but make decisions right away and act. In fact, a cross-cultural study has shown a more presence orientation and short-term planning in a Brazilian sample compared to a German sample with more pronounced long-term planning (Strohschneider and Güss, 1998).

## Limitations of the Thinking-Aloud Method

As with every method, the thinking aloud method has limitations. One problem might be related to the completeness of the data. First, some participants may not talk consistently and may remain quiet for long periods of time. The experimenter can remind participants briefly and unobtrusively to keep talking, but a few participants will not be able or willing to do so. It may even be that for participants of certain cultures it is unusual, uncomfortable, and unnatural to spontaneously say out loud what they think (see Kim, 2002). The author showed that East Asian Americans had different attitudes regarding talking and thinking aloud compared to European Americans. East Asian Americans regarded talking not as important when solving problems and indicated they talked less often at home with their parents compared to European Americans.

In some cultures, for instance, people learn to keep quiet and stay quiet until they have something worthwhile to say. So there might be some screening or sifting through what they say out loud. In fact, we found cross-cultural differences in the number of statements made during the 12 min of the simulations, *F*(4,386) = 23.47, *p* < 0.001, partial η^2^ = 0.196. For those tapes that were described and contained more than 10 statements, the average number of statements was 88 for Brazilians, 103 for Germans, 82 for U.S. Americans, 69 for Filipinos, and 49 for Indians. Future research will have to address the validity of the thinking-aloud method for various cultural groups. Potentially familiarizing participants with this method and having practice sessions before starting an experiment could be helpful.

Second, background noise or a participant with a very soft voice can make it hard to understand the participant’s verbal utterances on the tape. To instruct the participant to speak louder might affect the data. It is difficult for a person to change the volume of his or her voice. Speaking louder would most likely require conscious effort, which could possibly limit working memory capacity needed for focusing on the task.

Third, not every cognitive process is active in working memory and can be verbalized. Some psychological processes do not reach consciousness or are automatic processes that cannot be verbalized (Wilson, 1994). Fourth, sometimes a participant may experience various thoughts, but may not have the time to express all of them and, therefore, will be required to select what to report. The fifth limitation is a practical one. The analysis of thinking-aloud protocols is tedious, time consuming, and labor intensive.

An open question refers to the reactivity of thinking-aloud data in various cultures as well as the validity across cultures, as mentioned before. Ericsson and Simon (1993) have put together various studies on verbal reports in Western countries and have shown that it is a quite reliable and valid method if participants are not instructed or stimulated to observe their problem-solving processes and to engage in metacognitive activities that might in turn influence and redirect their problem solving or trigger new thought processes (reactive effects of verbal protocols). Future research will show whether thinking-aloud protocols are also a reliable and valid method of gathering data in non-western countries. Future research could also show for which processes and phenomena across cultures the thinking-aloud method is more and less useful.

## Conclusion

Thinking aloud refers to the concurrent verbalization of thoughts while performing a task. It is a method widely used in various areas of psychology, however, not in cross-cultural psychology. This paper discussed the limitations of the method and showed its strengths by discussing various opportunities for cross-cultural research: improving validity of cross-cultural surveys by investigating cultural meanings of survey items, investigating psychological processes rather than outcomes across cultures, testing theories cross-culturally, and allowing individual and group-level analyses across cultures. Thus, thinking-aloud protocols can offer additional insights in human minds around the world.

## Ethics Statement

This study was carried out in accordance with the recommendations of the Institutional Review Board of the University of North Florida with written informed consent from all subjects. All subjects gave written informed consent in accordance with the Declaration of Helsinki. The protocol was approved by the Institutional Review Board of the University of North Florida.

## Author Contributions

The author confirms being the sole contributor of this work and approved it for publication.

## Conflict of Interest Statement

The author declares that the research was conducted in the absence of any commercial or financial relationships that could be construed as a potential conflict of interest.
